# Detecting Malingered COVID‐19 Symptoms Using the Verifiability Approach

**DOI:** 10.1002/brb3.71278

**Published:** 2026-02-20

**Authors:** Raffaella Maria Ribatti, Tiziana Lanciano, Antonietta Curci

**Affiliations:** ^1^ Department of Education, Psychology, Communication Sciences University of Bari Aldo Moro Bari Italy; ^2^ Department of Bioscience, Biotechnology and Environment University of Bari Aldo Moro Bari Italy

**Keywords:** COVID‐19, malingering, noncredible symptom reports, symptom validity, verifiability approach

## Abstract

**Introduction:**

The COVID‐19 pandemic has heightened concerns about malingering, particularly given its recognition as an occupational disease in several regions. This study evaluated the applicability of the Verifiability Approach (VA), a credibility assessment tool based on the principle that liars provide fewer verifiable details than truth tellers, to distinguish between honest reporters and malingerers of COVID‐19 infection.

**Methods:**

A total of 410 participants (51.5% female) completed an online survey via Google Forms. Participants who reported previous COVID‐19 infection (*n* = 205) were assigned to either an *informed honest* (*n* = 104) or *not‐informed honest* (*n* = 101) group, while those without prior infection (*n* = 205) were assigned to *informed malingerers* (*n* = 105) or *not‐informed malingerers* (*n* = 100) conditions. Participants in the informed condition were briefed about the VA before writing their reports.

**Results:**

Informed honest participants provided significantly more verifiable details than both uninformed honest participants and malingerers. The number of verifiable details and the ratio of verifiable details to total details were strongly associated with honesty when participants were informed about the VA, indicating that the information Protocol enhanced diagnostic accuracy. Perceived success was higher among honest participants, particularly those informed about the VA. No significant effects emerged for reported long COVID or fabricated symptoms, likely due to limited variability and low symptom familiarity.

**Conclusion:**

The findings support the VA's validity in distinguishing genuine from feigned symptom reports in health‐related contexts. However, the fully online design, lack of factual verification, and potential for misreporting represent key limitations. Future studies should replicate these results in face‐to‐face or ecologically valid settings and extend the VA framework to the study of symptom dissimulation.

## Graphical abstract

Using the verifiability approach, this study shows that genuine COVID‐19 reports include more verifiable details than fabricated ones. Informing participants about the method further increases accuracy. Results highlight new possibilities for detecting deceptive symptom presentations across health‐related, clinical, and forensic settings.

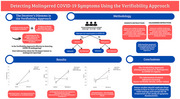

## 1 Introduction

Malingering is the deliberate fabrication or exaggeration of symptoms for external gains, such as avoiding work, legal consequences, or obtaining financial or drug‐related benefits (American Psychiatric Association 2013). It presents a significant challenge, costing over $5 billion annually in medical and legal expenses (Gouvier et al. 2003), and strains justice and mental health systems by diverting resources from those who genuinely need them (Rogers and Bender 2018; Resnick and Knoll 2018).

Malingering rates vary by context and method, from about 5% in clinical samples (Martin and Schroeder 2020; Schroeder et al. 2022) to over 50% in forensic settings (Chafetz et al. 2007; Grills and Armistad‐Jehle 2016). This behavior is not limited to offenders. Studies have shown that many individuals in the general population admit to feigning symptoms (Walczyk et al. 2018; Dandachi‐FitzGerald et al. 2020). For example, 34% reported doing so, often for minor ailments like headaches or fevers (Dandachi‐FitzGerald and Merckelbach 2013). Among psychology students, 41% admitted to malingering for academic or personal gain (Merten and Giger 2018; Boskovic 2020).

Common motives for malingering include taking sick leave (36.3%), receiving social security benefits (20.6%), filing insurance claims (15%), and taking extended vacations (11.9%; Puente‐López et al. 2023). Sick leave is the top reason among both the general population (42.4%) and students (38.1%; Puente‐López et al. 2023).

Detecting malingering remains challenging. Unstructured methods such as clinical interviews often fail to reliably identify deception, even among experienced professionals (Dandachi‐FitzGerald et al. 2017; Resnick and Harris 2002; Rosen and Phillips 2004). To improve accuracy, researchers have developed structured interviews (Rogers et al. 1992), self‐report screening tools (Smith and Burger 1997; Merten et al. 2016), response style analyses (Rogers and Vitacco 2002), technological methods (Monaro et al. 2018), and verbal credibility assessment techniques (Akehurst et al. 2017). Given the inherently deceptive nature of malingering, recent studies highlight the potential of integrating advances in lie detection (Walczyk et al. 2018).

### 1.1 Lessons From Verbal Deception Detection: The Verifiability Approach

Deception research has examined various cues, with verbal indicators proving more reliable than nonverbal ones, especially in high‐stakes settings (Bond and DePaulo 2006; Curci et al., 2019, Porter and ten Brinke 2010; Vrij 2008). Truth‐tellers typically give richer, more detailed accounts, while liars provide less specific information (Amado et al. 2016; DePaulo et al. 2003; Masip et al. 2005; Oberlader et al. 2016; Vrij 2005, 2008). Deceivers often add numerous details to appear credible but typically provide fewer genuine specifics due to the cognitive effort required for fabrication (Bell and Loftus 1989; Hartwig et al. 2007). To avoid contradictions, especially when verification is expected, they tend to omit verifiable false information (Masip and Herrero 2013; Nahari et al. 2014c).

The verifiability approach (VA; Nahari, 2018) builds on the observation that liars tend to avoid providing details that could be independently checked. It distinguishes between verifiable (details linked to specific people, places, or times) and unverifiable information. While truth‐tellers typically include both types of details in their accounts, liars are more likely to rely on unverifiable information to reduce the risk of being exposed (Strömwall et al. 2006; Vrij et al. 2010). Furthermore, explaining the VA through an “Information Protocol” improves its accuracy in detecting deception (Harvey et al. 2017a; Nahari et al. 2014b; Nahari and Pazuelo 2015; Vrij et al. 2016). Informed truth‐tellers increase verifiable details, while deceivers do not—likely due to a lack of genuine information. This makes the protocol a key element of the VA (Nahari 2019). The ratio of verifiable to total details reliably distinguishes liars from truth‐tellers, with higher ratios consistently linked to truthful accounts (Nahari et al. 2014b).

The VA has been successfully applied across multiple domains, including law enforcement (Nahari and Vrij; Nahari et al. 2014a, 2014b; Vernham et al. 2020), insurance claims (Harvey et al. 2017a; Harvey et al. 2017b; Nahari et al. 2014c; Vrij et al. 2016), airport security (Jupe et al. 2017; Kleinberg et al. 2017), occupational settings (Jupe et al. 2016), and malingering detection (Boskovic et al. 2017; 2019a; Boskovic et al. 2019b; Acka et al. 2020).

Research by Boskovic et al. 2017, 2019a, 2019b) on the VA in cases of noncredible pain and PTSD symptom presentations has yielded important insights. Their findings suggest that individuals feigning symptoms often provide a high number of unverifiable details but few that can be objectively confirmed. However, when malingerers were prompted with an information protocol, they produced longer statements than truth‐tellers by fabricating false verifiable details (Boskovic et al. 2017). This indicates that explicit warnings about verification might lead both genuine and feigned accounts to become equally elaborate, potentially complicating typical deception detection patterns.

Despite this, Nahari (2019) highlights the inherent challenges of applying the VA to malingering, particularly because many symptoms are intangible and lack direct documentation. Unlike observable physical signs, verifiable details often rely on recorded or witnessed evidence, which is inherently more challenging to obtain when symptoms are fabricated or subjective. However, symptom reports frequently include verifiable behaviors such as doctor visits or medication use (Boskovic et al. 2018), which can serve as objective points of reference. The concept of “compelling inconsistencies” (Bianchini et al. 2005) further underscores how verifiable evidence can expose deception. For instance, if a patient claiming disability is later observed working, and witnesses or official records corroborate this contradiction, it provides substantial proof of malingering—a phenomenon often highlighted in the media. While commonly feigned symptoms like fever, cough, or fatigue are subjective and difficult to verify directly, associated actions, such as doctor appointments, medication purchases, test results, or observable behaviors, can offer potential verifiable details (Sherman et al., 2020). This approach is particularly auspicious in the context of COVID‐19, where symptom exaggeration or fabrication can be cross‐referenced with verifiable medical records and testing data.

### 1.2 COVID‐19 and Long‐COVID Symptoms

COVID‐19, first identified in Wuhan, China, in late 2019, primarily causes respiratory symptoms like fever and cough. Declared a pandemic by the WHO in March 2020, it led to lockdowns and social distancing, which adversely affected mental health. These measures contributed to depression, sleep problems, anxiety, PTSD symptoms, memory issues, and emotional difficulties (Gualano et al. 2020; Brooks et al. 2020; Taylor et al. 2020; Lanciano et al. 2024; Mangiulli et al. 2022; Ribatti et al. 2022; Bendau et al. 2021; Qiu et al. 2021; Talevi et al. 2020; Wang et al. 2021; Zhang and Ma 2020). Fear took many forms, including separation anxiety and infection fears, sometimes causing obsessive behaviors and social withdrawal (Buccolo et al. 2020; Lanciano et al. 2020; Banerjee and Rai 2020). COVID‐19 patients show increased post‐traumatic stress, depression, and altered social perception compared to controls (Taurisano et al. 2022).

Studies have documented persistent neuropsychiatric symptoms, commonly referred to as “Long‐COVID,” including fatigue, cognitive dysfunction, sleep disturbances, and mood and anxiety disorders following the acute phase of the illness (Badenoch et al. 2022; Hampshire et al. 2021). Persistent symptoms often extend beyond a year post‐infection, featuring dysexecutive syndrome, inattention, and motor coordination issues (Helms et al. 2020). These cognitive impairments frequently co‐occur with anxiety and depression (Almeria et al. 2020). Essential workers, particularly healthcare personnel, have faced disproportionately high infection rates due to increased exposure risk (Ladhani et al. 2020; Baker et al. 2021; Carlsten et al. 2021). Other sectors, such as transportation and food services, also experienced significant exposure (Sears et al. 2020). Beyond the physical health impact, the pandemic has imposed significant psychological stress on workers, potentially impairing work productivity (AronssHernández et al. 2021; Jamal et al. 2021). Consequently, understanding trends in absenteeism and presenteeism is critical, with factors such as working conditions, job satisfaction, and employee well‐being playing pivotal roles in addressing these challenges (Barmby et al. 2002; Beemsterboer et al. 2009; Livanos and Zangelidis 2013; Lusinyan and Bonato 2007).

Research has shown an increase in sick leave among healthcare workers during the pandemic (Calvo‐Bonacho et al. 2020). COVID‐19's classification as an occupational disease (Carlsten et al. 2021) has raised concerns about malingering. In Italy, INAIL officially recognizes COVID‐19 infection as a workplace injury under Decree Law No. 18 (March 17, 2020), equating it with accidents. Marinaccio et al. (2020) found that 19.4% of cases were workplace‐acquired, primarily in high‐risk sectors such as healthcare, social care, meat processing, retail, postal services, pharmacies, and cleaning. Additionally, LeGoff et al. (2023) assessed the utility of neurocognitive screening in managing post–COVID–19 symptoms and facilitating employees' return to work. Although malingering was not the primary focus, the study revealed that 48% of participants provided invalid responses and demonstrated noncredible effort on psychological and cognitive assessments.

The ambiguity and persistence of symptoms complicate the validation of health claims, thereby increasing the risk of symptom exaggeration or fabrication, which may be aimed at prolonging sick leave or obtaining benefits. Therefore, addressing malingering in the context of COVID‐19 is critical to distinguishing genuine health concerns from false claims.

## 2 Methods

### 2.1 Aims and Hypotheses

The present study aims to evaluate the effectiveness of the verifiability approach (VA; Nahari et al. 2014a, 2014b) in accurately detecting malingering of COVID‐19 infection. Given the global impact of COVID‐19 and the limited empirical research on malingering related to it, understanding potential symptom exaggeration or feigning for external gains is crucial. This study addresses this gap and explores innovative methods for malingering detection.

Building on the work of Nahari et al. (2014a, 2014b), Nahari and Pazuelo (2015), and Boskovic et al. (2017, 2018, 2019a), we conduct a comparative analysis between self‐reports of individuals who contracted COVID‐19 (honest participants) and those instructed to simulate infection (malingerers). We also examine whether informing participants about the VA mechanism helps distinguish between truthful and deceptive reports (Nahari et al. 2014b). Additionally, participants were asked about their experiences with genuine or simulated long‐COVID symptoms.

Consequently, the hypotheses are formulated as follows:

H1: Informed honest participants are expected to report a greater number of verifiable details compared to both not‐informed honest participants and both instructed and not‐instructed malingerers. Conversely, malingerers will not demonstrate significant differences in the endorsement of verifiable information.
H2: Informed honest participants will provide a higher ratio of verifiable details to total details compared to other conditions.
H3: Informed honest participants would report a higher perceived success in convincing the experimenter they were honest compared to both informed and not‐informed malingerers.
H4: Malingerers would report a higher number of bogus long‐COVID symptoms compared to honest participants.

### 2.2 Design and Participants

We used G*Power (Faul et al. 2007) to conduct a priori power analysis for an ANOVA with four groups, assuming *α* = 0.05, a power of 0.95, and an effect size of *f* = 0.25, which corresponds to a sample size of approximately 400 participants. An initial sample of 420 participants was recruited for the study. After an initial screening, 10 participants were excluded from the study due to admitting to malingering or providing insufficient effort in their descriptions (fewer than five details). A total sample of 410 participants (51.5% women) was recruited. The average age of the sample was 33.11 years (*SD *= 12.68, range = 18–67), with an average level of education of 14.20 years (*SD *= 2.62; range = 8–22). For further details, see Table 1.

**TABLE 1:** Description of the study population.

Variable	Total sample	Informed honests	Not‐informed honests	Informed malingerers	Not‐ informed malingerers
*N* (%)	410 (100%)	104 (25.4%)	101 (24.6%)	105 (25.6%)	100 (24.4%)
Women, *n* (%)	211 (51.5%)	55 (52.9%)	52 (51.5%)	53 (50.5%)	51 (51%)
Age, M (SD)	33.11 (12.68)	29.66 (9.84)	33.00 (11.97)	34.27 (14.68)	35.55 (13.21)
Education (years), M (SD)	14.20 (2.62)	14.32 (2.12)	14.71 (2.37)	13.64 (2.28)	13.61 (2.57)

The study was conducted between July 2022 and January 2023. The entire procedure was conducted online using a Google Forms survey. Initially, the researchers collected data from a cohort of 200 participants sourced from the experimenters' acquaintances based on the scrutinizing questions. Subsequent participants were then recruited via social networks (Facebook and Instagram) and stratified based on their self‐reported COVID‐19 status to ensure an even distribution across both honest and malingerer categories.

The study was approved by the Ethical Committee of the University of Bari and conducted in accordance with the Declaration of Helsinki (No. ET‐21‐16).

### 2.3 Materials and Procedure

The study employed a 2 × 2 between‐subjects design with condition (honest vs. malingerer) and information (informed vs. not‐informed) as factors, and the number of verifiable details and the verifiable details/total details ratio as dependent variables.

A detailed description of the experimental protocol is provided in Supporting Information S 1. Our methodology replicated Nahari et al. (2014b) with adjustments: the study was conducted in Italy (not Israel), without additional incentives for credible statements, and participants responded in Italian. Although no strict time limit was imposed, participants completed the task within 15 min. We introduced an exclusion criterion for noncompliance with instructions and included a post‐task question assessing participants' perceived success (see Nahari et al. 2014) in convincing the experimenter they were honest, serving as a manipulation check given the challenge deceivers face in fabricating convincing lies (Walczyk et al. 2014). Finally, participants were asked about the experience of genuine or simulated long‐COVID symptoms.

Upon providing informed consent and answering some demographic questions, participants accessed the study via a Google Forms link while the experimenter was connected via video call.

First, participants were asked whether they had ever tested positive for COVID‐19. Those who disclosed having experienced COVID‐19 (*n* = 205) were randomly categorized into two groups: informed honest (*n* = 104) and not‐informed honest (*n* = 101). At the same time, those reporting that they never had COVID‐19 (*n* = 205) were randomly assigned to the conditions of informed honests (*n* = 105) and not‐informed malingerers (*n* = 100).

Participants who reported a prior COVID‐19 infection were instructed to compose an email to their physician detailing all symptoms experienced during the acute phase and any persistent symptoms related to long COVID‐19 to obtain a medical certificate for sick leave. They were also explicitly asked to provide precise information about the contagion circumstances, including medications taken, contacts during infection, and activities while infected. Participants denying prior COVID‐19 exposure completed a similar task but were asked to fabricate the information.

Those in the informed condition received an information protocol statement before the task, warning that their details might be verified (cf. Harvey et al.2017a, 2017b; see Supporting Information S3). After submitting their report, participants rated their perceived success in convincing the experimenter on a 1‐to‐7 scale. Finally, participants indicated whether they experienced (genuine or fabricated) long‐COVID symptoms (yes/no) and were shown a list of commonly reported long‐COVID symptoms from WHO (2020), alongside several bogus symptoms never documented in the literature (e.g., lack of thirst, loss of sight, genital herpes, nail fungus; see Supporting Information S2).

Upon completing the task, participants received a debriefing that explained the true purpose of the study. Throughout, responses were mandatory before proceeding, and participants could withdraw at any time.

### 2.4 Data Analyses

All simple effects and post hoc comparisons were conducted in *R* (version 4.4.1; *R* Core Team, 2^2023^) using the packagesfor the ANOVA and *emmeans* for the estimation of marginal means and pairwise contrasts. To control for Type I error, the Bonferroni correction was applied to all pairwise post hoc comparisons. Effect sizes (partial *η* ^2^) were computed using the *effectsize* package. The reported *p* values in Section 3 are adjusted accordingly.

Before conducting the main analyses, the distributional properties of the dependent variables were assessed. Skewness (< 1.4) and kurtosis (≤ 2.3) indicated moderate but acceptable deviations from normality. According to simulation studies such as those of Curran et al. (1996), univariate skewness below 2 and kurtosis below 7 seldom distort parameter estimates or Type I error rates, particularly with large, balanced samples. Consistent with these findings and further evidence supporting ANOVA's robustness to moderate non‐normality (Blanca et al. 2017; Schmider et al. 2010), the assumption of approximate normality was considered satisfied.

**FIGURE 1:**
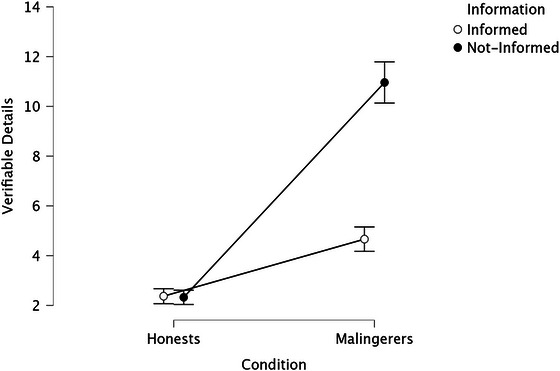
Interaction effect of information × condition on the number of verifiable details.

**FIGURE 2:**
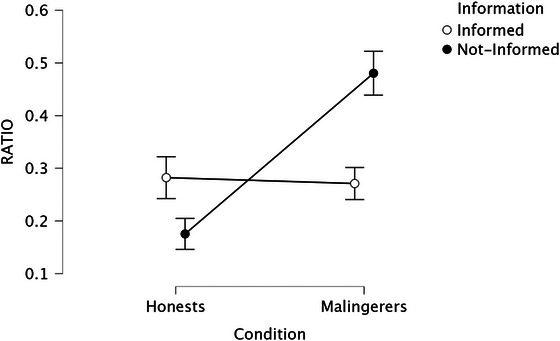
Interaction effect of information × condition on the ratio scores.

**FIGURE 3:**
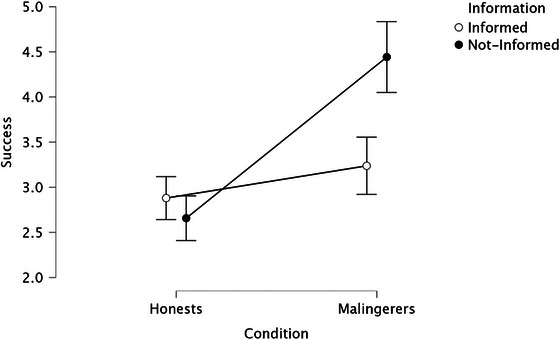
Interaction effect of information × condition on the success scores.

Preliminary descriptive statistics were computed for each dependent variable. A series of two‐way analyses of variance (ANOVAs) was conducted with *information* (informed vs. not informed) and *condition* (honest vs. malingerers) as between‐subject factors. Separate ANOVAs were performed for (a) the total number of details, (b) the number of verifiable details, (c) the ratio of verifiable details to total details, (d) perceived success in the task, and (e) the number of reported long‐COVID symptoms. For each ANOVA, *F*‐values, *p* values, and *η_p_* ^2^ effect sizes were reported. The significance threshold was set at *α* = 0.05 (two‐tailed). Where significant interactions were detected, simple effects analyses were conducted to examine the direction of the effects. Descriptive statistics and group means are presented in the corresponding tables (Tables 2, 3, 4), and significant interactions are graphically illustrated in Figures 1, 2, 3.

**TABLE 2:** Descriptive statistics of the number of total details by information and condition.

Measures		Mean (*SD*)
Information	Informed	20.11 (11.27)
	Not‐informed	14.06 (9.45)
Condition	Honest	22.22 (11.90)
	Malingerers	12.07 (6.50)
Information* Condition	Informed honest	25.33 (12.32)
	Informed malingerers	14.94 (7.05)
	Not‐informed honest	19.02 (10.59)
	Not‐informed malingerers	9.06 (4.11)

**TABLE 3:** ANOVA for the number of verifiable details by information and conditions.

Measures		Mean (SD)	*F*_(1, 406)_	*p*	Partial *η^2^*
Information	Informed	6.62 (5.37)	138.23	<0.001	0.25
	Notinformed	3.52 (2.35)			
Condition	Honest	7.86 (4.71)	422.56	<0.001	0.51
	Malingerers	2.35 (1.50)			
Information* Condition	Informed honest	10.96 (2.67)	142.34	<0.001	0.26
	Informed malingerers	2.32 (1.48)			
	Not‐informed honest	4.66 (2.48)			
	Not‐informed malingerers	2.37 (1.51)			

**TABLE 4:** ANOVA for the ratio scores by information and condition.

Measures		Mean (SD)	*F*_(1, 406)_	*p*	Partial *η^2^*
Information	Informed	0.28 (0.18)	8.101	0.005	0.02
	Not informed	0.33 (0.24)			
Condition	Honest	0.38 (0.21)	66.702	<0.001	0.14
	Malingerers	0.23 (0.19)			
Information* Condition	Informed honest	0.48 (0.21)	77.264	<0.001	0.16
	Informed malingerers	0.18 (0.15)			
	Not‐informed honest	0.27 (0.15)			
	Not‐informed malingerers	0.28 (0.20)			

### 2.5 Coding Details

The coding methodology followed the established guidelines as delineated in prior research (Nahari et al., 2014b) and was adapted to malingering applications (Akca et al.^2020^). The criteria for details to be categorized as verifiable included: (a) the details being documented and thus checkable (e.g., appointments with a doctor, prescriptions); (b) details involving actions carried out with identified individuals rather than alone or with non‐traceable strangers (e.g., visiting the doctor with a parent); (c) details relating to events witnessed by other identified individuals (e.g., calling a doctor in the presence of someone else); (d) details reported as being recorded (e.g., on CCTV; e.g., entering a pharmacy); (e) the use of technology (e.g., phone, tablet, or computer; e.g., searching symptoms online, making a doctor's appointment); or (f) details that can potentially be verified through blood analysis and/or medical tests (e.g., taking specific pills; Boskovic et al. 2018; Nahari & Vrij, 2014). The coding process was conducted by two independent coders who were blinded to the experimental conditions and tasked with evaluating all reports. Specifically, the total count of details (“total details”) and the count of verifiable details (“verifiable details”) were recorded. Any disparities between coders were resolved to reach a final consensus on each detail. The inter‐coder agreement, assessed using Cohen's Kappa, demonstrated a notably high level of concordance, yielding coefficients of 0.86 (*p* < 0.001) for total details and 0.89 (*p* < 0.001) for verifiable details.

## 3 Results

### 3.1 Total Details Descriptives

For a more comprehensive approach, we conducted a preliminary analysis of the descriptive statistics for Total details before proceeding to the ANOVA (see Table 2).

The descriptive statistics reveal that Honest participants reported more total details than malingerers. Informed honest reported the highest number of total details, while not‐informed malingerers reported the lowest number of total details.

### 3.2 Number of Verifiable Details

We conducted an analysis of variance (ANOVA) with information and condition as factors and the number of verifiable details as the dependent variable. For completeness, descriptives and main effects are reported in Table 3.

Results revealed a significant main effect of information, indicating that participants who were informed provided more verifiable details than those who were not informed. Additionally, a significant main effect of condition was observed, indicating that Honest participants provided more verifiable details than malingerers.

The information × condition interaction effect was statistically significant (see Figure 1).

Specifically, simple effects show that when participants were in the honest condition, those who were informed provided a significantly higher number of verifiable details compared to those who were not informed, *F*_(1, 406)_ = 538.21, *p* < 0.001, *η_p_* ^2^ = 0.57. Conversely, when participants were malingerers, there was no significant difference between the Informed and not‐informed groups in the number of verifiable details reported (*M* = 2.37, SD = 1.52), *F*_(1, 406)_ = 0.02, *p* = 0.90, *η_p_* ^2^ = 0.001. Additionally, when participants were informed, Honest individuals reported significantly more verifiable details than malingerers, *F*_(1, 406)_ = 280.61, *p* < 0.001, *η_p_* ^2^ = 0.41. The same pattern emerged in the not‐informed condition, although the effect was smaller, *F*_(1, 406)_ = 36.49, *p* < 0.001, *η_p_* ^2^ = 0.08.

### 3.3 The Ratio of Verifiable Details/Total Detail Score

We conducted an analysis of variance (ANOVA) with information and condition as factors, and the ratio of verifiable details to total details score (ratio) as the dependent variable. For completeness, descriptives and main effects are reported in Table 4.

The analysis revealed a significant main effect of information, indicating that participants who were informed had higher ratio scores than those who were not. Furthermore, there was a significant main effect of condition, indicating that honest participants provided higher ratio scores than malingerers.

The interaction effect between information and condition was statistically significant (see Figure 2). Follow‐up simple effects analyses showed that, among honest participants, the effect of information was significant, *F*_(1, 406)_ = 67.71, *p *< 0.001, *η_p_* ^2^ = 0.14, with informed honest participants providing a greater proportion of verifiable details than not‐informed honest participants. Among malingerers, the effect of information was also significant, *F_(_*_1, 406)_ = 17.66, *p* < 0.001, *η_p_^2^ *= 0.04, indicating that informed malingerers provided higher ratio scores than not‐informed malingerers. Conversely, the effect of condition was significant only among informed participants, *F*_(1, 406)_ = 146.63, *p *< 0.001, *η_p_* ^2^ = 0.27, showing that informed honest reported markedly higher ratio scores than informed malingerers. Among not‐informed participants, the effect of condition was not significant, *F*_(1, 406)_ = 0.19, *p* = 0.66, *η_p_* ^2^< 0.001.

### 3.4 Post‐Report Questions

#### 3.4.1 Self‐Perceived Success

We conducted an analysis of variance (ANOVA) with information and condition as factors and perceived success as the dependent variable.

Results showed a significant main effect of information. Informed participants (*M* = 3.55, *SD* = 1.92) perceived themselves as having been more successful than not‐informed participants (*M* = 3.06, *SD* = 1.42; *F*_(1, 406) _= 10.125, partial *η_p_* ^2^ = 0.02, *p* < 0.005).

A main effect of condition was also observed, indicating that honest participants perceived themselves as more successful in convincing the experimenter (*M* = 3.85, SD = 1.92) compared to malingerers (*M* = 2.77, SD = 1.24; *F*_(1, 406)_ = 48.22, *η_p_^2^* = 0.11, *p* < 0.001).

The interaction effect between information and condition was also significant, *F*_(1, 406)_ = 21.40, *p *< 0.001, *η_p_* ^2^ = 0.05. Follow‐up simple effects analyses showed that, among nformed participants, honest individuals (*M* = 4.44, *SD* = 2.02) perceived themselves as having been significantly more successful than malingerers (*M* = 2.66, *SD* = 1.28), *F*_(1, 406)_ = 68.27, *p* < 0.001, *η_p_^2^* = 0.14. Among not‐informed participants, this difference was not significant, *F*_(1, 406)_ = 2.64, *p* = 0.11, *η_p_^2^* = 0.006. When examining the effect of information within each condition, results indicated that informed honest participants reported higher self‐perceived success than not‐informed honests, *F*_(1, 406)_ = 30.49, *p* < 0.001, *η_p_^2^* = 0.07. In contrast, among malingerers, the effect of information was not significant, *F*_(1, 406)_ = 1.04, *p* = 0.31, *η_p_^2^* = 0.003.

#### 3.4.2 Long‐COVID Questions

We conducted an analysis of variance (ANOVA) with information and condition as factors and the sum of reported long‐COVID symptoms as the dependent variable. Results showed that neither the main effect nor the interaction was significant, *F* < 1.70, *ps* > 0.05, *ηs_p_^2^* < 0.012. Another ANOVA was conducted with information and condition as factors, and the sum of reported long‐COVID fake symptoms as the dependent variable. Analyses showed no significant main or interaction effect, *F* < 1.03, *ps* > 0.05, *ηs_p_^2^* < 0.003.

## 4 Discussion

The present study aimed to examine the applicability of the verifiability approach (VA) in distinguishing honest reporters from malingerers within the context of COVID‐19 and long COVID symptoms. Specifically, it compared symptom reports provided by individuals who had genuinely experienced COVID‐19 (honests) with those instructed to simulate the condition (malingerers), while also assessing whether prior information about the VA influenced participants’ reporting strategies.

A preliminary examination of total details showed that honest participants generally provided longer and richer accounts than malingerers, confirming that genuine recollection tends to produce more elaborate narratives. This effect was particularly strong among informed, honest participants, who produced the most extensive reports overall. These findings are consistent with prior deception research demonstrating that truth‐tellers include more details because they rely on actual episodic memory, while deceivers, drawing on fabricated or reconstructed accounts, tend to simplify their narratives to minimize the risk of inconsistencies (Vrij 2008; Nahari 2019). However, this result diverges from previous findings in the field of malingering and VA, where total detail count has often failed to discriminate between liars and truth‐tellers (Boskovic et al. 2017, 2018). One possible explanation lies in the different nature of the task: while previous studies involved simulated mental or physical disorders, our participants described a discrete and temporally bounded event (COVID‐19 infection). Such experiences are more likely to evoke vivid episodic memories, enabling honest individuals to recall numerous contextual details, whereas malingerers may rely on schematic or generalized representations of the illness. Thus, the higher total detail production observed in honest participants may reflect the episodic specificity of the COVID‐19 experience rather than a generalizable effect of honesty across all clinical contexts.

As expected, informed participants provided more verifiable details than uninformed participants, and honest individuals outperformed dishonest ones, in line with previous research (Nahari 2019). Specifically, receiving the information protocol notably increased the number of verifiable details among honest participants, while malingerers were largely unaffected. This interaction suggests that the VA mechanism amplifies the natural differences in reporting style between genuine and simulated accounts, supporting its diagnostic potential in malingering contexts, in that honest individuals benefit from the instruction, while malingerers cannot effectively imitate the strategy.

Regarding the ratio of verifiable to total details, informed participants displayed higher ratios than uninformed participants, and honest participants, especially when informed, consistently outperformed those who were not honest. These findings support Hypotheses 1 and 2 and align with prior research (Nahari et al. 2014a, 2014b; Palena et al. 2021), confirming that verifiable details may be used as reliable indicators of honesty. The pattern also highlights that informing malingerers about the VA slightly increased their ratio scores, suggesting partial awareness of the strategy, yet their performance remained significantly below that of honest participants, consistent with the idea that deception constrains the inclusion of verifiable information (Vrij et al. 2021). Nevertheless, our findings diverge from those of previous malingering studies in the VA realm (Akca et al. 2020; Boskovic et al. 2017, 2018), where the number of verifiable details did not emerge as a significant marker of honesty in symptom reporting. These discrepancies may stem from differences in how the VA was applied. Unlike previous work that used generalized symptom lists or chronic conditions, the present study focused on a discrete, temporally bounded event (COVID‐19), where symptoms and experiences were more easily anchored to verifiable facts such as testing dates or quarantine locations. This may have enhanced the VA's sensitivity in detecting fabricated reports.

Analyses of perceived success reinforced these findings: informed honest participants reported feeling significantly more successful than all other groups, including both informed and uninformed malingerers as well as uninformed honest participants. This aligns with Hypothesis 3 and resonates with the results from Nahari et al. (2014b), indicating that deceivers face greater challenges in appearing credible compared to honest participants. Informed malingerers’ lower perceived success may also reflect increased awareness of their limited ability to fabricate verifiable information once the verification principle was introduced.

The low number of reported long‐COVID symptoms limited the statistical analyses. This outcome may reflect that honest participants had not actually experienced such symptoms, whereas malingerers may have deliberately avoided mentioning them to appear more credible (Nahari et al. 2012). Given the relative rarity and ambiguity of long COVID during the data collection period, simulators may have preferred to describe more familiar and recognizable COVID‐related symptoms. Consequently, Hypothesis 4 could not be empirically verified due to the inconsistency and scarcity of relevant data. Nonetheless, this null finding underscores the importance of symptom familiarity and perceived plausibility in shaping deceptive reporting strategies.

While these findings highlight the potential of the verifiability approach (VA) in detecting malingering and the role of information provision in eliciting more comprehensive and verifiable details, several limitations should be acknowledged.

The first limitation concerns the methodological nature of the study, which was conducted entirely online through a Google Forms platform. Research indicates that deception tends to be easier in written, asynchronous contexts than in face‐to‐face settings, where nonverbal cues and anxiety levels are more salient (Eskritt et al., 2025). The lack of direct interaction might therefore have reduced the emotional engagement typically associated with deceptive behavior. Future studies should aim to replicate the present design in controlled, in‐person conditions to evaluate how communication mode and social presence influence the production of verifiable details.

A second limitation pertains to the operationalization of “verifiable details.” Participants were asked to include elements that could, in principle, be externally verified (e.g., names, places, specific events), yet no factual verification of these details was carried out. This methodological choice reflected the study's main objective, that is, to examine the *production* of potentially verifiable content rather than its actual accuracy, in accordance with previous VA research (e.g. Nahari et al., 2014b,^2019^ . However, the absence of an independent verification process prevents conclusions about the factual truthfulness of participants’ statements. Because the data were collected online, verifying personal information would also have been impractical and potentially raised privacy and ethical concerns. Future studies could adopt partial verification procedures or ethically feasible corroboration methods to enhance the ecological validity of the VA in applied contexts.

A further limitation lies in the characteristics of the illness investigated. COVID‐19 symptoms share several features with common flu‐like conditions, which could facilitate the fabrication of plausible symptom reports. This familiarity may limit the generalizability of our findings to illnesses that are less well known or more complex (e.g., Leins et al. 2013; Nahari, 2018a; Vrij 2008). Future research could, therefore, apply the VA to rarer or less familiar medical conditions, focusing on secondary‐level verifiable information to test the robustness of the approach across different symptom domains.

The absence of monetary compensation or tangible incentives represents another limitation. Although this choice minimized potential demand effects, it also reduced ecological validity, as real‐life malingering is typically motivated by concrete external rewards, such as financial gain, avoidance of responsibility, or access to resources. This experimental design, commonly adopted in deception research (e.g., Rogers and Cruise 1998), allows for controlled comparisons but may not fully capture the motivational and emotional factors that characterize real‐life malingering scenarios. Consequently, participants’ engagement and deceptive strategies may differ from those of actual malingerers encountered in clinical or forensic settings. Future studies could integrate more ecologically valid paradigms by including participants with genuine incentives to feign or by replicating the procedure in applied contexts, such as medico‐legal or occupational health evaluations.

Another limitation concerns the possibility that some participants misreported their COVID‐19 infection history in the preliminary questionnaire. Given the social and political sensitivity surrounding the pandemic (e.g., vaccination debates, stigma, and differing beliefs about severity), it cannot be ruled out that some individuals, intentionally or unintentionally, provided inaccurate information. Such misreporting could have influenced group assignment and the interpretation of results. Although screening questions were implemented to minimize this risk, self‐reported data remain vulnerable to bias. Future studies should consider incorporating objective verification measures, such as requiring medical documentation or test results, to ensure diagnostic reliability.

A specific limitation concerns the inconclusive findings regarding Hypothesis 4 on long COVID symptoms. The scarcity and inconsistency of these reports constrained statistical power and interpretability. This likely reflects both the low prevalence of long COVID among honest participants and the tendency of malingerers to avoid mentioning unfamiliar or ambiguous symptoms in order to appear credible. Furthermore, at the time of data collection, public understanding of long COVID was still emerging and fragmented, leading to variability in symptom conceptualization. Future research could address this issue by employing more targeted prompts or recruiting verified long COVID patients to ensure sufficient variability and ecological validity.

Finally, it is plausible, that during the pandemic, some individuals were more motivated to *dissimulate* than to *exaggerate* symptoms; that is, to pretend to be healthy rather than ill. Under strict public health restrictions, concealing symptoms could allow individuals to maintain employment, social life, or freedom of movement, thereby functioning as an adaptive deceptive strategy. For example, Boskovic et al. (2024) found that 14% of participants admitted to feigning a COVID‐19 infection, mainly to stay home or obtain sick leave, while 12% reported concealing an actual infection to avoid stigma or missing events. Younger individuals were more likely to hide illness, and when asked about people they knew, these rates nearly doubled (28% for feigning and 51% for concealment), suggesting that symptom misrepresentation occurred on a meaningful scale and may have biased self‐reported COVID‐19 data. Meanwhile, widespread media exposure provided individuals with abundant and coherent information about COVID‐19 symptoms, offering both models for credible malingering and cues for strategic dissimulation (Leins et al. 2013). This dynamic suggests that deception during health crises may operate bidirectionally, encompassing both the overreporting and underreporting of symptoms. Extending the verifiability approach to study dissimulation could, therefore, clarify whether individuals who conceal illness similarly limit verifiable content to avoid detection (Nahari 2018). Despite these limitations, the present study has the potential to contribute to the growing literature on the VA by demonstrating its applicability to symptom reporting contexts and by highlighting the moderating role of information provision in distinguishing honest from deceptive narratives.

## 5 Conclusions

The present study marks an innovative foray into the realm of detecting malingering amid COVID‐19. By leveraging the VA to scrutinize the provision of details and verifiable information, this research extends our understanding of malingering behaviors associated with COVID‐19 symptoms. Although the study has its limitations, notably the familiarity with COVID‐19 symptoms, its pioneering nature lays the groundwork for further exploration. The insights gleaned from this investigation hold substantial implications across diverse domains.

Hence, the outcomes of this study present a valuable tool that could aid in detecting fabricated information related to COVID‐19 symptoms, not only in healthcare but also in settings where individuals might exploit the pandemic to shirk responsibilities, such as the workplace.

## Author Contributions

**R.M.R**.: conceptualization, methodology, investigation, data curation, formal analysis, writing – original draft preparation. **A.C**.: supervision, validation. **A.C**. and **T.L**.: methodology, data curation, writing – reviewing and editing, visualization.

## Funding

The authors have nothing to report.

## Ethics Statement

The study was approved by the University of Bari and conducted in accordance with the Declaration of Helsinki (Approval No. ET‐21‐16).

## Consent

Informed consent was obtained from all participants involved in the study.

## Conflicts of Interest

The authors declare no conflicts of interest.

## Supporting information




**Supplementary Material**: brb371278‐sup‐0001‐SuppMat.docx

## Data Availability

The data that support the findings of this study are available from the corresponding author upon reasonable request.
