# Supplementary material for: Detecting Malingered COVID‐19 Symptoms Using the Verifiability Approach
Source: Brain Behav. 2026 Feb 20;16(2):e71278. doi: 10.1002/brb3.71278 (PMC12922456; doi:10.1002/brb3.71278)
Supplement: Supplementary file 1 — Supplementary Material: brb371278‐sup‐0001‐SuppMat.docx [file BRB3-16-e71278-s001.docx]

**Supplemental materials**

### **S1. Experimental Protocol**

#### **Phase 1 – Screening and Group Assignment**

1. **Screening question: “Have you ever tested positive for COVID-19?”**
   - If **Yes** → assigned to the Honest condition
     Randomly divided into:
     - **Informed Honest group** (N = 104)
     - **Not-Informed Honest group** (N = 101)
   - If **No** → assigned to the Malingerer condition
     Randomly divided into:
     - **Informed Malingerer group** (N = 105)
     - **Not-Informed Malingerer group** (N = 100)

#### **Phase 2 – Main Task (Written Report)**

1. **General instruction:**
   - “You are asked to compose an email to your physician describing your experience with COVID-19, as if you were requesting a medical certificate for sick leave.”
2. **Specific task instructions:**
   - **Honest participants (those who had COVID-19):**
     - “*Please write an email to your physician describing your experience with COVID-19 infection. In your message, include all details about the symptoms you experienced, the circumstances of infection, any treatments or medications taken, and your daily activities during the illness. The email should be written as if you were requesting a medical certificate for sick leave.”*
   - **Malingerers (those who never had COVID-19):**
     - “Please write an email to your physician, imagining an episode of COVID-19 infection. In your message, include details about the symptoms you experienced, the circumstances of infection, any treatments or medications taken, and your daily activities during the illness. Use your imagination to make the description as realistic and detailed as possible, as if you were requesting a medical certificate for sick leave”

#### **Phase 3 – Information Protocol (Informed vs. Not-Informed Conditions)**

1. **Information protocol (subsequent instructions)**
   - **Informed condition:** Participants were presented with an **Information Protocol Statement** (adapted from Boskovich and colleagues' (2016) study)
     - **“**Research indicates that deceptive individuals tend to avoid reporting details that can be confirmed, whereas honest ones lean towards providing verifiable Information. Consequently, we will present your statement to healthcare professionals, seeking their evaluation of its accuracy based on the verifiability of the details you provide. Verifiable details encompass activities that can be documented and verified, such as phone calls, medical appointments, prescriptions, etc., or activities that can be corroborated through blood analysis and medical records. These should involve interaction with another identifiable person or be witnessed by someone identifiable, and CCTV cameras could also record them. Details that do not meet these criteria are classified as unverifiable. Therefore, when reporting your symptoms, ensure to maintain their verifiability by providing Information that demonstrates their authenticity. Please note that the information you provide may be subject to verification. Ensure that your answers are accurate and consistent.”
   - **Not-Informed condition:**
     - No warning or additional statement was provided before the task.

#### **Phase 4 – Self-Rating and Symptom Check**

1. **Self-assessment of performance:**
   - “On a scale from 1 to 7, how successful do you think you were in convincing the experimenter with your report?”
     (1 = not convincing at all, 7 = extremely convincing)
2. **Self-report on Long-COVID symptoms:**
   - “Do you experience any Long-COVID symptoms (either genuine or fabricated)?”
     - Options: Yes / No
3. **Symptom checklist:**
   - Participants were shown a **list of symptoms** (check S2 – Supplemental Materials) including:
     - Genuine Long-COVID symptoms (from WHO, 2020)
     - Bogus symptoms (never reported in the literature; e.g., lack of thirst, loss of sight, genital herpes, nail fungus)
   - Task: “Please indicate which of the following symptoms you experienced.”

#### **Phase 5 – Debriefing**

1. **Debriefing statement:**
   - Participants were informed of the **true purpose** of the study (to investigate deception and malingering in symptom reporting).
   - They were reminded that their participation was voluntary and that they could withdraw their data if they wished.

**S2. The List of long-COVID-19 symptoms from the WHO website (2020) was presented to participants, with the non-COVID symptoms indicated in italics.**

1. Fatigue
2. Fever
3. Muscle Aches
4. Joint Aches
5. Respiratory Difficulties
6. Cough
7. Tachycardia
8. Chest Pain
9. *Arrhythmia*
10. *Murmur*
11. Hypertension
12. Headache
13. Brain Fog
14. Memory Problems
15. Insomnia
16. Loss of taste and smell
17. Neuropathies
18. Anxiety
19. Depression
20. *Mood Disorders*
21. *Obsessions*
22. Abdominal Pain
23. Vomiting
24. Diarrhea
25. *Reflux*
26. *Allergies*
27. Tinnitus
28. Throat Pain
29. Dysphonia
30. *Swollen tongue*
31. *Hemorrhoids*
32. Skin Eruptions
33. Rash
34. *Urticaria*
35. Hair loss
36. *Nail Fungi*
37. *Loss of Thirst*
38. *Hypersalivation*
39. *Thyroiditis*
40. *Loss of sight*
41. *Genital Herpes*
42. *Itching hands*

**S3. Examples of reports, with details separated by "/" and verifiable details in bold.**

1. **Informed truth-teller code: G*****5**

**Details: 58**

**Verifiable details: 22**

On the morning /of 27/01/2022/, following the news received from / **my partner /** regarding his COVID-19 positivity/, I decided to contact **my primary care doctor** / **via text message**. He instructed me to go to the local pharmacy/, as they offered a drive-through testing service / in the adjacent parking lot/. I then put on my mask / and went **to the local pharmacy**,/ and after about ten minutes from the swab /(performed both in the nose / and throat), **I was informed of my positive status/**. Despite experiencing some symptoms such as cough /and sore throat/, I initially thought it was just a typical seasonal flu / and certainly not COVID-19/. The idea of being confined at home until I tested negative / completely destabilized me!/ **I tested positive** for / **17 days** /, while **my partner** / **only for 7**/, and **his colleague** / **for slightly more**/, so I assume my partner / was the source of this chain of infections! / **The doctor/ prescribed some pills/** like **azithromycin**/ and **cortisone**/. I / immediately / bought an oximeter / **on Amazon** / and underwent the treatment./ After 17 days/, **I took the / third / test** / and it was wonderful /to know that **I was no longer contagious and infected**!/

I had spent / all those days / in bed / or on the couch **/ or in the garden / with the dog** /, **despite video calls / and received calls** /. It was a continuous succession of identical days/ where the only changes were the pajamas/ or the movie /, all confined to a room/ or the garden/.

1. **Non-informed truth-teller: C*****5**

**Details: 45**

**Verifiable details: 9**

I began to suspect that I had contracted COVID-19 in the /last days /of December/ 2020/, after learning that some family members/, with whom I had been in contact in the preceding days**/,** had tested positive/. I received confirmation of a positive result **after undergoing the swab test on** January 3, 2021./ After receiving the result,/ a report was filed /with the **local health authority** /(ASL), which monitored the situation/ and **scheduled control swabs** /week by week/. I was afraid /of how the situation might evolve/, fearing the worst/ as we were at the beginning of the second wave/ and there was no talk of vaccines yet./ Fortunately, everything went well!/ I tested positive for a total of 4 weeks,/ **receiving a negative result**/ on January/ 27/, 2021/. As symptoms, I only had a mild cold /treated with anti-inflammatory medication, /and a week after the positive swab,/ I started to lose my sense of taste and smell/. I thought taste and smell would return after a maximum of a few weeks,/ but it actually took almost a year /for them to come back. /To try to recover my taste and smell,/ on **the doctor's advice**,/ I started **taking Be-Total** /along with **Nicetile**,/ but there was no improvement. /I had almost lost hope,/ but with a specific supplement, **/Assonal**, /I gradually began to regain them.

1. **Informed malingerer: S*****7**

**Details: 31**

**Verifiable details: 5**

I tested positive for Covid-19/ on June/ 27th /of this year (2022). /After spending a sleepless night /with flu-like symptoms/, cough,/ sore throat, /muscle aches/, and nausea, /the next morning, /I decided to take a swab test./ **I went to the pharmacy** /**with my aunt** /and **mother** /(yes, they had also shown symptoms related to the virus)/, and I found out that **I had been infected**/. I'm not sure where I might have contracted it,/ but probably at work/, as I come into contact with many different people every day./ I experienced the isolation period in a fairly/ comfortable/ room/, having everything I needed. /Luckily, **I live in a two-floor house**, /so I didn't have contact with my family,/ avoiding the potential risk of infecting them./ I tested positive for a week /and only needed Tachipirina, /especially initially./ Fortunately, I didn't have symptoms that persisted /even after testing negative.

1. **Non-informed malingerer:** P*****9

**Details: 30**

**Verifiable details: 5**

About a month ago/, I found out I tested positive for COVID-19, /. It was around 1:00 PM /when, after lunch,/ I started feeling a pain in my chest/ and a sensation of a lump in my throat/. Concerned about what happened**,/ I immediately called/ my mother**/, who advised me to get a test /. As the hypochondriac that I am, /I rushed to **the pharmacy**/ and immediately took the test, /which turned out to be undoubtedly **positive./**

I was quite worried/ because I had never experienced this illness before/, and I didn't know how to react./ The pains persisted,/ and anxiety took over /as I started feeling a general discomfort /and an expanding sense of anxiety within me./ I tested positive for about two weeks/, but the illness evolved positively/. The pains decreased, /and my breathing improved/. Only yesterday did I finally find out that I am no longer positive, /although **my doctor** advised me to continue with the prescribed treatment /for at least this week, /consisting of supplements/ and other medications./
